# Impact of chemotherapy and immunotherapy on survival in pediatric primary diffuse leptomeningeal melanomatosis: a systematic review and pooled survival analysis

**DOI:** 10.1007/s00381-026-07264-2

**Published:** 2026-05-01

**Authors:** Khushi H. Shah, Mauricio J. Guerrero-Ocampo, Italo G. Flecha, Caleigh S. Roach, Sima Vazquez, Diego Servián, Victor M. Lu

**Affiliations:** 1 Department of Neurological Surgery, University of Miami Miller School of Medicine, Miami, FL USA; 2 Hospital de Clinicás, Universidad Nacional de Asunción, San Lorenzo, Paraguay; 3 Department of Neurological Surgery, Hospital General Pediátrico “Niños de Acosta Ñu”, San Lorenzo, Paraguay

**Keywords:** Pediatric, Primary diffuse leptomeningeal melanomatosis, Primary melanocytic tumors, Meningeal melanomatosis

## Abstract

**Purpose:**

Pediatric primary diffuse leptomeningeal melanomatosis (PDLM) is a rare, aggressive malignancy with no established standard of care. We performed an integrated survival analysis to characterize disease course and identify treatment factors associated with overall survival (OS).

**Methods:**

A systematic literature search of PubMed, Embase, and Scopus (2000–2025) was conducted in accordance with PRISMA guidelines. Eligible studies included patients < 18 years with histopathologically confirmed PDLM per 2021 World Health Organization and Expert Care for Rare Adult Solid Cancers criteria and reported OS and treatment data. Survival was assessed using Kaplan–Meier analyses, with univariable and multivariable Cox proportional hazards regression.

**Results:**

Thirty-three pediatric PDLM cases were identified (57.6% male; median age 10 [IQR 3–14] years). Diagnosis was established via tissue sampling in 72.7%, cerebrospinal fluid cytology in 21.2%, and both in 6.1%. Among 13 patients with molecular testing, 69.2% harbored NRAS mutations. Twenty-four patients (72.7%) received treatment: chemotherapy (70.7%), radiation therapy (41.7%), and immunotherapy (62.5%). Median OS was 5.0 [3.5–7.0] months. On Kaplan–Meier analysis, immunotherapy was associated with improved OS (*p* < 0.001), including when combined with radiation (*p* = 0.038), chemotherapy (*p* = 0.006), or both (p = 0.048). Univariable Cox regression showed immunotherapy to be significantly associated with reduced hazard of death (HR 0.23 [0.10–0.51], *p* < 0.001). On multivariable analysis, immunotherapy (HR 0.22 [0.10–0.49], *p* < 0.001) and chemotherapy (HR 0.44 [0.21–0.94], *p* = 0.035) were independently associated with improved OS.

**Conclusion:**

Pediatric PDLM carries a uniformly poor prognosis. Immunotherapy and chemotherapy were the only treatments independently associated with improved survival, highlighting their central role in management of this rare disease.

## Introduction

Primary melanocytic tumors of the central nervous system (CNS) arise from leptomeningeal melanocytes and account for approximately 1% of all melanocytic neoplasms [1, 2]. Within the WHO classification, these lesions range from benign to malignant entities and may present as discrete intradural masses or diffuse leptomeningeal disease [2].

Pediatric primary diffuse leptomeningeal melanomatosis (PDLM) represents the most aggressive end of this spectrum and constitutes an exceptionally rare subset of pediatric CNS tumors [2, 3]. Since its initial descriptions in the nineteenth century [4, 5], the literature has remained limited largely to isolated case reports, with contemporary reviews identifying only small pediatric cohorts ranging from 5 to 15 pediatric PDLM cases [6–10].

PDLM follows a fulminant clinical course characterized by rapidly progressive neurologic decline and early mortality. Children typically present with nonspecific symptoms of increased intracranial pressure, and diagnosis requires histopathologic or cytologic confirmation with diffuse leptomeningeal involvement in the absence of systemic melanoma [11]. Treatment guidance remains limited, as chemotherapy, radiotherapy, and immunotherapy have yielded inconsistent results, and no modality has demonstrated a clear survival advantage in ultra-rare CNS melanocytic tumors [12]. Importantly, pediatric PDLM has not been examined separately, leaving major gaps in understanding treatment responsiveness in children.

To address these gaps, we performed a systematic review of published pediatric PDLM cases from the past 25 years and conducted pooled survival analyses. To our knowledge, this represents the largest contemporary aggregation of pediatric PDLM. Our objective was to characterize clinical presentation, radiographic and CSF features, molecular alterations, treatment patterns, and survival outcomes, and to assess whether any therapeutic modality is associated with improved prognosis in this ultra-rare malignancy.

## Methods

### Case identification

Institutional Review Board (IRB) approval and patient consent was waived given that data was collected from publicly available sources. A systematic search of PubMed, Embase, and Scopus was then conducted in September 2025 in accordance with Preferred Reporting Items for Systematic Reviews and Meta-Analyses (PRISMA) guidelines [13] and registered in the PROSPERO database (PROSPERO 2026 CRD420251249660). To maximize sensitivity, Boolean operators to search permutations containing the terms “pediatric primary diffuse leptomeningeal melanomatosis,” “primary leptomeningeal melanoma,” and “pediatric melanocytic tumor” were used. Reference lists of retrieved articles were manually screened for additional cases.

### Selection criteria and data extraction

Eligible cases included patients < 18 years of age with histopathologic confirmation consistent with the 2007 WHO criteria for PDLM, radiographic evidence of diffuse leptomeningeal involvement, documentation of treatment details, and reported overall survival. Articles published prior to the 2007 WHO classification were re-evaluated and recategorized when possible. Exclusion criteria were unclear diagnosis, adult patients, secondary leptomeningeal melanoma, duplicate cases, insufficient clinical or survival data, non-English publications, and review articles.

Titles and abstracts were independently screened by two authors (KS and MG), with discrepancies resolved by a third reviewer (CR). Full texts meeting inclusion criteria underwent standardized data extraction into a predefined spreadsheet. The final list of articles included are listed in Table 1. Extracted variables included demographics, presenting symptoms, neurologic examination, hydrocephalus, imaging findings, diagnostic methods, histopathology and immunohistochemistry, CSF findings, molecular alterations, treatments received, and survival outcomes.

**Table 1:** Studies included in integrated cohort (*n *= 33 cases)

Case No	Study	Year	Publication type	Country	Age at diagnosis (years)	Gender	Follow-up (months)	Final status
1	Raja et al., 2003	2003	Article	United States	11	M	16	D
2	Chu et al., 2003	2003	Article	China	3	F	7	D
3	Comstock et al., 2009	2009	Article	United States	0.75	F	3.5	D
4	Subbiah et al., 2009	2009	Article	United States	4	F	6	A
5	Hayes-Jordan et al., 2011	2011	Article	United States	3	F	9	D
6	Berzero et al., 2015	2015	Article	Italy	17	M	0.5	D
7	Szathmari et al., 2016	2016	Article	France	5	F	11	D
8	Angelino et al., 2016	2016	Article	Italy	2.25	F	11	D
9	Green et al., 2017	2017	Abstract	United Kingdom	0.5	F	4	D
10	Chen et al., 2019	2019	Article	China	1.6	M	4	D
11	Schaff et al., 2019	2019	Article	United States	16	M	3	D
12	Hung et al., 2018	2018	Article	Taiwan	6	F	1.5	D
13	Xu et al., 2020	2020	Article	China	13	M	5	D
14	Yagi et al., 2020	2020	Abstract	Japan	4	M	4	A
15	Ke et al., 2021	2021	Letter to Editor	China	16	F	3	D
16	Tavana Rad et al., 2021	2021	Article	Iran	14	F	6	D
17	Baumgartner et al., 2021	2021	Article	Austria	14	M	7	D
18	Quinn et al., 2021	2021	Article	United States	3	M	5	D
19	Quinn et al., 2021	2021	Article	United States	15	M	1	D
20	Pezzullo et al., 2021	2021	Article	Italy	16	M	5	D
21	Pezzullo et al., 2021	2021	Article	Italy	14	M	5	D
22	Pezzullo et al., 2021	2021	Article	Italy	15	M	1	D
23	Mastronuzzi et al., 2022	2022	Article	Italy	14	F	6	D
24	Yamauhci et al., 2022	2022	Article	Japan	14	M	18	D
25	Kumar et al., 2024	2024	Article	India	12	M	4	D
26	Marquez et al., 2024	2024	Article	Argentina	14	F	4	D
27	Takahashi et al., 2024	2024	Article	Japan	2	F	6	D
28	Shahab et al., 2024	2024	Article	United States	3	M	7	D
29	Oren et al., 2024	2024	Abstract	Israel	13	M	9	D
30	Oren et al., 2024	2024	Abstract	Israel	7	M	12	D
31	Zuo et al., 2025	2025	Article	China	8	M	1	D
32	Sudharsan et al., 2025	2025	Article	India	7	F	5	D
33	Sareh et al., 2024	2024	Article	Iran	10	M	3	D

*F* female, *M* male, *D* dead, *A* alive

### Statistical analysis

Categorical variables were summarized as counts and percentages, while continuous variables were reported as medians with interquartile ranges (25th-75th percentile). Kaplan–Meier analyses estimated overall survival (OS), with comparisons made using the log-rank test. Univariable Cox proportional hazards testing was performed on variables with 10 or more events. Variables with a univariable association of *p* < 0.10 were utilized for multivariable modeling to identify independent factors associated with hazard of death. Multicollinearity was assessed for final model to ensure VIF < 5. All tests were two-sided, with statistical significance defined as *p* < 0.05. Analyses were performed using Python (version 3.11.5, macOS) and GraphPad Prism (version 10.1.2; GraphPad Software Inc.).

## Results

**Fig. 1:**
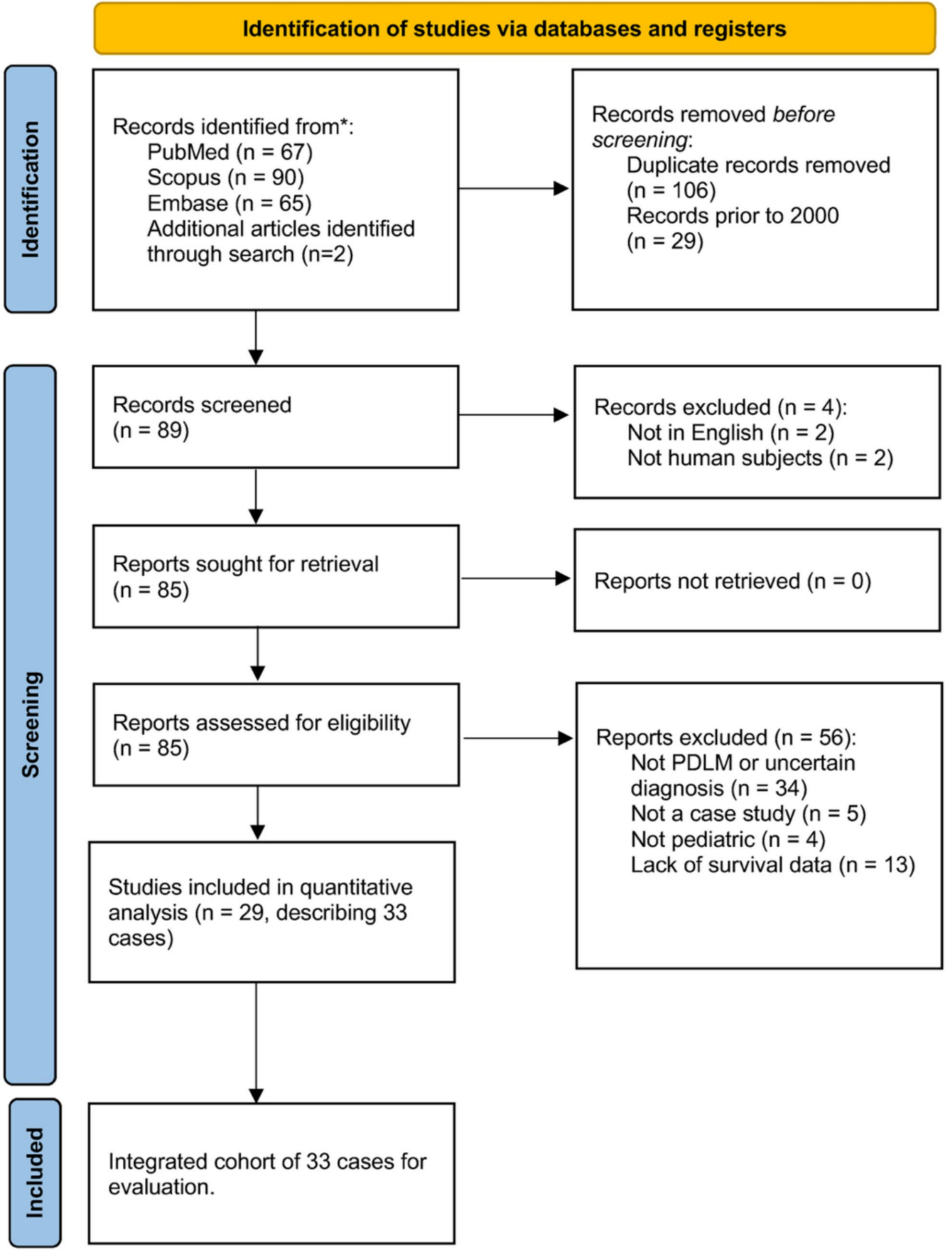
PRISMA diagram depicting process of identification of cases included in integrated analysis

The search identified 224 articles. After removing 106 duplicates and 29 published before 2000, 89 abstracts were screened. Eighty-five articles underwent full-text review, yielding 29 studies reporting 33 pediatric PDLM cases (Fig. 1). Median age at diagnosis was 10 (IQR 3–14) years, and 57.6% of patients were male. Median OS was 5 (IQR 3.5–7) months, and 93.9% of patients had died by last follow-up.

### Clinical presentation

Headache (54.5%) and nausea/vomiting (72.7%) were the most frequent presenting symptoms. Additional neurologic findings included visual deficits (27.3%), motor deficits (22.2%), seizures (12.1%), and cranial nerve abnormalities (6.1%) (Table 2).

**Table 2:** Patient demographics, presentation, diagnosis, and treatment details

Variable	Integrated cohort (*n* = 33)	Variable	Integrated cohort (*n* = 33)
Patient demographics		Negative for genetic mutations	4/13 (30.8%)
Age, years	10 [3-14]	**CSF diversion**	*n* = 22/32 (68.8%)
Gender, male	19/33 (57.6%)	VP shunt	18 (81.8%)
Clinical presentation		Ommaya reservoir	4 (18.2%)
Headache	18/33 (54.5%)	EVD	1 (4.5%)
Nausea/vomiting	24/33 (72.7%)	**Oncologic treatment, yes**	24/33 (72.7%)
Motor deficit	8/33 (24.2%)	**Chemotherapy**^**2**^	*n* = 17/24 (70.8%)
Cranial nerve palsies	2/33 (6.1%)	Temozolomide-based regimens	11 (64.7%)
Visual deficit	9/33 (27.3%)	Platinum based agents	5 (29.4%)
GCS < 8 on initial arrival	2/33 (6.1%)	Cisplatin	4 (23.5%)
Seizure	4/33 (12.1%)	Carboplatin	1 (5.9%)
Cutaneous findings		Alkylating agents	4 (23.5%)
Giant nevus (> 10 cm)	6/23 (26.1%)	Dacarbazine	3 (17.7%)
Smaller nevi	5/23 (21.7%)	Carmustine	1 (5.9%)
Imaging findings		Cyclophosphamide	2 (11.8%)
CT	10/33 (30.3%)	Antimetabolites	3 (17.7%)
MRI	32/33 (93.9%)	Methotrexate	1 (5.9%)
Hydrocephalus	12/33 (36.4%)	Cytarabine	2 (11.8%)
Diffuse leptomeningeal enhancement	33/33 (100%)	Topoisomerase inhibitors	2 (11.8%)
Parenchymal infiltration	16/32 (50.0%)	Topotecan	1 (5.9%)
Parenchymal lesion	11/32 (34.4%)	Etoposide	2 (11.8%)
Lesion involvement^1^	*n* = 11	Microtubule inhibitors	4 (23.5%)
Frontal lobe	3/10 (27.3%)	Vincristine	1 (5.9%)
Parietal lobe	2/10 (18.2%)	Vinblastine	1 (5.9%)
Temporal lobe	3/10 (27.3%)	Vindesine	2 (11.8%)
Intraventricular	2/10 (18.2%)	Not reported	3 (17.7%)
Corpus callosum	1/10 (9.1%)	**Radiation therapy**^**2**^	*n* = 10/24 (41.7%)
Cerebellum	1/10 (9.1%)	Whole brain	2 (20.0%)
Spinal	1/10 (9.1%)	SC	6 (60.0%)
Diagnosis		Focal	2 (20.0%)
CSF cytology only	7/33 (21.2%)	Not reported	1 (10.0%)
Tissue diagnosis only	24/33 (72.7%)	**Immunotherapy**^**2**^	*n* = 15/24 (62.5%)
Biopsy	20/33 (60.6%)	Checkpoint inhibitor	7 (29.2%)
Surgery	3/33 (9.1%)	Nivolumab	7 (29.2%)
Biopsy followed by surgery	1/33 (3.0%)	Ipilimumab	5 (20.8%)
Both cytology and tissue diagnosis	2/33 (6.1%)	BRAF inhibitor — vemurafenib	1 (4.2%)
Macroscopic pigment visualization	17/19 (89.5%)	Tyrosine kinase inhibitor — sorafenib	2 (8.3%)
Immunohistochemistry^2^	*n* = 20	MEK inhibitor	3 (12.5%)
HMB-45	16/20	Trametinib	1 (4.2%)
S-100	10/20	Binimetinib	1 (4.2%)
Melan-A	12/20	Exact drug not specified	1 (4.2%)
Genetic testing	*n* = 13	Type I interferon therapy	3 (12.5%)
NRAS mutation	9/13 (69.2%)	mTOR inhibitor — everolimus	3 (12.5%)

^1^Sum of subtotals exceeds count of patients due to some patients with lesions with involvement of multiple structures

^2^Sum of subtotals exceeds the number of treated patients due to multiple agents being used

Cutaneous melanocytic markers were inconsistently reported. Giant congenital melanocytic nevi were identified in 6/24 patients (26.1%) with 5 patients with concomitant smaller nevi. Small nevi alone were present in 1 patient.

### Hydrocephalus and CSF diversion

Hydrocephalus occurred in 36.4% of patients. Most patients (68.8%) ultimately required CSF diversion: ventriculoperitoneal shunting (*n* = 17), Ommaya reservoir (*n* = 3), external ventricular drainage (*n* = 1), or combination of VP shunt and reservoir implantation (*n* = 1).

### Neuroimaging

All patients demonstrated diffuse leptomeningeal enhancement on MRI or CT scan, establishing this as a hallmark feature. On MRI, this was most consistently identified on T1-weighted post-contrast sequences. CT imaging was performed in 30.3% of cases, while MRI was obtained in 93.9%. Parenchymal infiltration was identified in half the patients with 11 exhibiting discrete parenchymal lesions: frontal (*n* = 3), temporal (*n* = 3), and parietal lobes (*n* = 2), intraventricular lesions (*n* = 2), corpus callosum (*n* = 1), cerebellum (*n* = 1), and spinal cord (*n* = 1).

### Diagnostic methods and histopathology

Diagnosis was established by tissue sampling in 72.7% of cases, CSF cytology in 21.2%, and both in 6.1%. Of the studies that mentioned details on histology, all tumors demonstrated high mitotic activity (*n* = 24) and cytologic atypia (*n* = 25). Macroscopic pigment was noted intraoperatively in 17 of 19 patients (89.5%). Immunohistochemistry (*n* = 20) consistently confirmed melanocytic origin, with tumors expressing HMB-45, Melan-A, and S-100 (Table 2).

### CSF characteristics

Initial LP findings were frequently abnormal but often nondiagnostic, with 15/23 (65.2%) of patients having a nondiagnostic initial LP. Among these, 5 patients had two nondiagnostic LPs and 1 patient had four nondiagnostic LPs. Of the patients with initial non-diagnostic LP, 80% (12/15) underwent tissue biopsy or surgery for definitive diagnosis.

### Molecular and genetic findings

Genetic testing was performed in 13 patients (38.2%). NRAS mutations were predominant (*n* = 9, 69.2%), often involving canonical hotspots such as Q61 or G12. One patient exhibited GNAQ R183Q and S1PR3 G89S mutations in CNS lesions and NRAS G12V in metastatic abdominal disease.

### Treatment patterns

Overall, 72.7% of patients received at least one form of oncologic therapy, with multimodal regimens used in 13/24 (54.2%). These included chemotherapy and immunotherapy only (*n* = 4), immunotherapy and radiation therapy only (*n* = 3), chemotherapy and radiation therapy only (*n* = 1), and triple therapy combining chemotherapy, radiation therapy, and immunotherapy (*n* = 5). Chemotherapy was administered in 70.8% of patients and involved temozolomide-based regimens (*n* = 11), including platinum-based agents (*n* = 5), alkylating agents (*n* = 4), and microtubule inhibitors (*n* = 4). Radiation therapy was used in 41.7% of patients, including craniospinal irradiation (*n* = 6), whole brain radiation (*n* = 2), or focal treatment (*n* = 2). Immunotherapy or targeted therapy was given to 62.5% of patients, including checkpoint inhibitors such as nivolumab and ipilimumab (*n* = 7), MEK inhibitors such as trametinib and binimetinib (*n* = 3), type I interferon therapy (*n* = 3), tyrosine kinase inhibitor sorafenib (*n* = 2), mTOR inhibitor everolimus (*n* = 2); and BRAF inhibitor vemurafenib (*n* = 1) (Table 2).

### Survival outcomes

The median OS was 5.0 months (IQR 3.5–7.0), with estimated OS of 75.8% at 3 months, 35.1% at 6 months, 17.5% at 9 months, and 7.0% at 12 months**.**

**Fig. 2:**
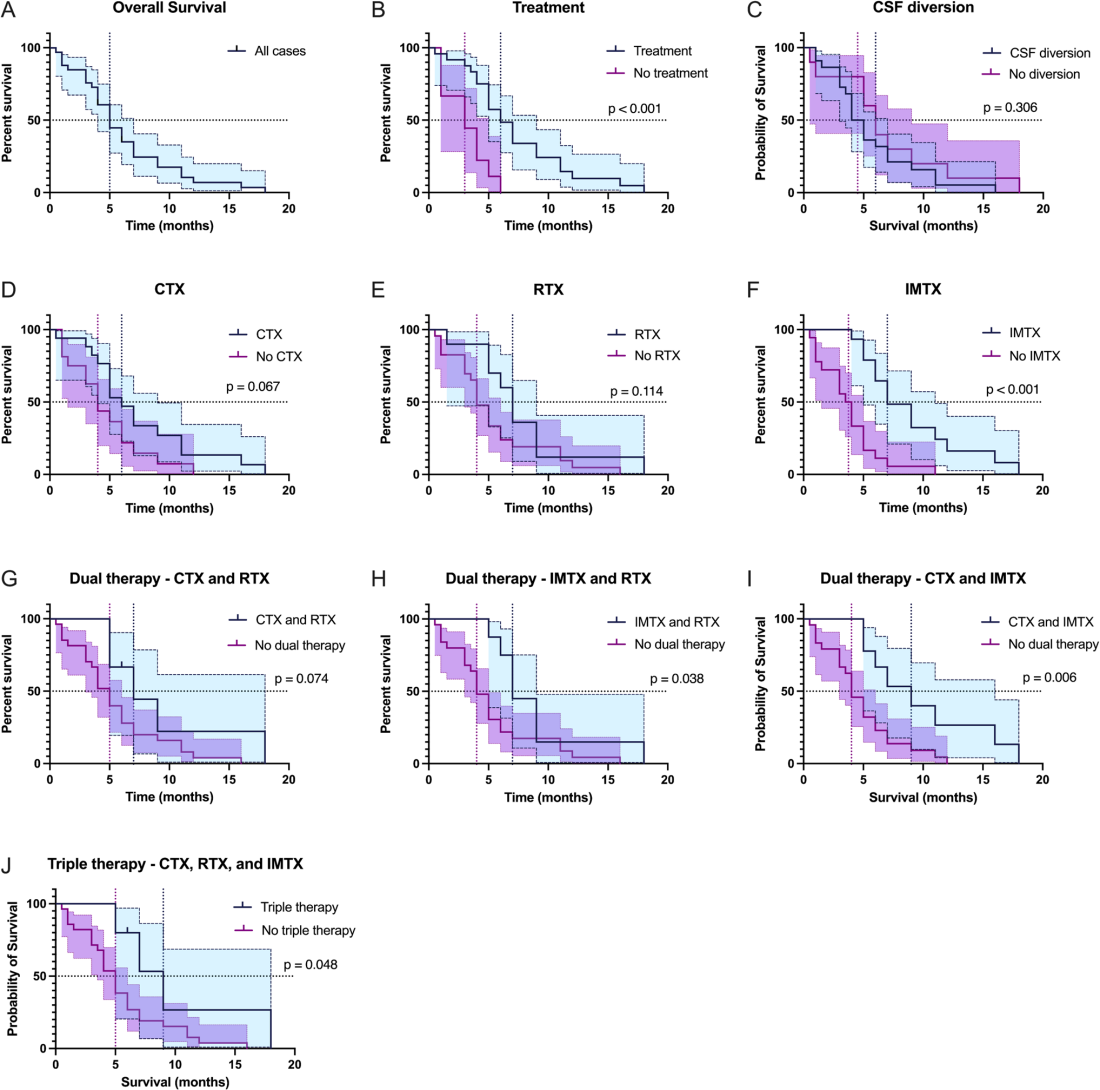
Kaplan–Meier plots of OS in **A**) all cases; **B**) any treatment; **C**) CSF diversion procedures; **D**) chemotherapy (CTX); **E**) radiation therapy (RTX); **F**) immunotherapy (IMTX); dual therapy with **G**) CTX and RTX; **H**) IMTX and RTX;** I**) CTX and IMTX; and **J**) triple therapy with CTX, RTX, and IMTX

Kaplan Meier analyses demonstrated improved survival with any form of oncologic treatment (median OS: 3 vs 6 months, *p* < 0.001), immunotherapy (3.75 vs 7 months, *p* < 0.001), combined immunotherapy and radiation therapy (4 vs 7 months, p = 0.038), combined chemotherapy and immunotherapy (4 vs 9 months, p = 0.006), or triple therapy (5 vs 9 months, p = 0.048). While patients who received chemotherapy (6 vs 4 months) and radiation therapy (4 vs 7 months) had improved median OS, the difference was not statistically significant (p = 0.067 and p = 0.114, respectively) (Fig. 2).

Upon univariate Cox regression, only immunotherapy significantly decreased hazard of death (HR 0.23, 95% CI [0.10–0.51], *p* < 0.001). Multivariate analysis identified both immunotherapy and chemotherapy as independent predictors of decreased mortality (HR 0.21, 95% CI [0.09–0.48], *p* < 0.001 and HR 0.46 [0.21–0.99], p = 0.047, respectively) (Table 3).

**Table 3:** Demographic and clinical parameters from presentation assess for predictors of OS by univariate and multivariate Cox regression

Variable	Univariable analysis		Multivariable analysis	
	Hazard ratio	*p* value	Adjusted hazard ratio	*p* value
**At diagnosis**				
Age	1.03 [0.96–1.11]	0.361		
Sex, male	0.98 [0.46–2.06]	0.952		
Headache	1.27 [0.61–2.62]	0.521		
Nausea/vomiting	1.40 [0.60–3.27]	0.441		
Hydrocephalus	1.00 [0.47–2.11]	0.993		
Parenchymal lesion	1.02 [0.48–2.16]	0.951		
**Treatment**				
CTX	0.52 [0.25–1.08]	0.081	0.46 [0.21–0.99]	**0.047**
RTX	0.53 [0.21–1.30]	0.163		
IMTX	0.23 [0.10–0.51]	** < 0.001**	0.21 [0.09–0.48]	** < 0.001**
CSF diversion	1.80 [0.84–3.87]	0.134		

Bold entries signify *p* < 0.05

*OS* overall survival, *CTX* chemotherapy, *RTX* radiation therapy, *IMTX* immunotherapy, *CSF* cerebrospinal fluid

## Discussion

This integrated cohort reinforces pediatric PDLM as an aggressive leptomeningeal malignancy characterized by diffuse neuroaxis involvement, rapid neurological decline, and poor survival. By aggregating all available pediatric cases, this study enables a more detailed characterization of clinical presentation, diagnostic challenges, molecular features, treatment patterns, and outcomes.

### Clinical presentation and delay in diagnosis

Pediatric PDLM most often presents with signs of increased intracranial pressure, consistent with prior reviews across all age groups [9]. Headache, nausea, and vomiting were common in our cohort, likely reflecting disruption of CSF dynamics from diffuse leptomeningeal infiltration [9]. Complications such as papilledema, seizures, and hydrocephalus have been described and were also observed in our case [11].

These symptoms are nonspecific and frequently mimic infectious or inflammatory conditions. Prior reports document misdiagnoses including tuberculosis, autoimmune encephalitis, meningitis, subarachnoid hemorrhage, and vascular syndromes, often leading to delayed or inappropriate treatment [7, 8, 14–18]. Cutaneous melanocytic lesions were present in only a minority of cases, limiting their diagnostic utility.

Given diagnostic complexity, clinicians should maintain a high index of suspicion for PDLM in children presenting with unexplained signs of increased intracranial pressure and diffuse leptomeningeal enhancement, even in the absence of cutaneous findings. Baumgartner et al. reported a median 3-month delay from symptom onset to diagnosis, which may further worsen prognosis [9].

### Radiologic presentation

All patients demonstrated diffuse leptomeningeal enhancement on neuroimaging, a hallmark feature of PDLM. Nearly half the patients also exhibited parenchymal infiltration or discrete lesions, underscoring its pan-neuroaxis behavior and the importance of whole-neuroaxis MRI at diagnosis.

### CSF characteristics and diagnostic challenges

CSF analysis was frequently abnormal, typically showing elevated opening pressure, pleocytosis, high protein, or low glucose, though initial cytology was commonly nondiagnostic [9]. Many patients required multiple LP with some still requiring tissue biopsy for definitive diagnosis. Baumgartner et al. noted that malignant cells were sometimes identified only after repeated sampling, emphasizing that a negative cytology does not exclude PDLM when clinical and radiographic findings are suggestive [9]. The diffuse growth pattern further complicates tissue sampling and may result in false-negative biopsies [19, 20]. Together, these findings reinforce that nondiagnostic CSF results do not rule out PDLM and that early neurosurgical biopsy, along with repeated CSF evaluation when feasible, is critical to avoid diagnostic delay.

Moreover, while CSF cytology can identify malignant melanocytic cells, its sensitivity is limited and results should be interpreted in the context of clinical and radiographic findings rather than in isolation. Emerging molecular approaches, including CSF-based genomic analysis such as detection of NRAS mutations via liquid biopsy, have been described in a small number of cases.

### Histopathology and immunophenotype

Tumors consistently demonstrated high mitotic activity, marked cytologic atypia, and macroscopic melanin pigment. Rare amelanotic variants have been reported and may complicate diagnosis [21]. Immunohistochemistry typically confirmed melanocytic origin with expression of HMB-45, Melan-A, and S-100.

### Molecular landscape

Among patients who underwent genetic testing, NRAS mutations were the most common, typically involving canonical oncogenic hotspots such as Q61 or G12 [6, 9, 15, 22]. NRAS activation has been linked to malignant progression in neurocutaneous melanosis and appears in both primary lesions and extracranial metastases [22]. Less commonly, GNAQ and other signaling-pathway mutations were identified [22].

Although molecular data were available for fewer than half of cases, the predominance of NRAS mutations suggests biological overlap with other NRAS-mutant melanocytic disorders. Importantly, NRAS alterations can occur even without cutaneous lesions, supporting their diagnostic value in ambiguous presentations [15]. These findings also raise the possibility that pathway-directed therapy, including MEK inhibitors, may have a role, although clinical experience remains limited [6].

### Management of hydrocephalus

Hydrocephalus typically develops in the later stages of PDLM [23]. In our review, nearly 70% of patients required CSF diversion procedures. However, CSF diversion remains palliative and does not alter disease trajectory, reinforcing the need for effective systemic or intrathecal therapies. Additionally, shunting carries a risk of intraperitoneal dissemination of malignant cells [6, 22]. Our institutional patient required two shunt revisions, consistent with reports of frequent obstruction due to high CSF protein and tumor-cell burden. Some authors have recommended incorporating inline filters within the shunt system to reduce distal obstruction or metastatic seeding [24].

### Overall survival

We observed a median OS of 5.0 (IQR 3.5–7.0) months. This is consistent with Baumgartner et al., who reported a 4-month median survival from diagnosis and 10 months from symptom onset in their review of 26 pediatric and adult patients with PDLM [9].

### Treatment patterns

Treatment strategies were heterogeneous and frequently multimodal, reflecting the absence of standardized protocols and the aggressive biology of the disease. Although PDLM is generally regarded as resistant to conventional chemotherapy and radiotherapy [16], our integrated analysis identified immunotherapy and chemotherapy as the only modalities with a reproducible survival benefit. Both were independently associated with improved OS in multivariable models, extending median survival from 3.75 to 7 months with immunotherapy and from 4 to 6 months with chemotherapy. These findings contrast with the EURACAN task force review of ultra-rare CNS melanocytic tumors, which concluded that no definitive systemic treatment advantage could be determined due to reliance on case reports and small cohorts [12].

Given the demonstrated efficacy of checkpoint inhibitors in extracranial melanoma and untreated melanoma brain metastases [25], similar benefit in PDLM is plausible. Immunotherapy produced the strongest survival association in our cohort, with nivolumab and ipilimumab being the most commonly used agents.

Chemotherapy also reduced hazard of death on multivariable analysis. Temozolomide-based regimens predominated, often combined with cytotoxic or intrathecal agents due to their CNS penetration. Evidence from secondary leptomeningeal melanomatosis suggests that intrathecal chemotherapy may prolong survival (HR 0.5, 95% CI [0.4–0.8], p = 0.004) [26]. Several authors incorporated intrathecal therapy for PDLM [9], hypothesizing that continuous intraventricular administration of alternating agents yielded higher leptomeningeal drug concentrations, resulting in rapid cytologic clearance and symptomatic improvement [9]. However, responses remain short-lived and progression common.

Radiation therapy did not demonstrate a survival benefit in our integrated analyses. This may reflect heterogeneity in dose, timing, indication, or incomplete reporting. EURACAN similarly recommends reserving high-dose radiation for palliation rather than disease modification [12].

Exploratory Kaplan Meier analyses suggest that incorporating immunotherapy in dual therapy regiments (immunotherapy and radiation or immunotherapy and chemotherapy) and triple therapy incorporating chemotherapy, radiation therapy and immunotherapy, may offer a survival benefit (*p* < 0.05 for all). Although interpretation remains constrained by small sample sizes, these findings raise the possibility that more aggressive, multimodal systemic treatment approaches could improve outcomes in pediatric PDLM.

### Limitations

This study has several important limitations. The cohort is small and composed primarily of individual case reports and small series, introducing heterogeneity in diagnostic evaluation, treatment approaches, and reporting quality. Not all clinical, radiographic, histopathologic, or molecular data were available for every patient, limiting analytical depth and the power of multivariable modeling. Publication bias is likely, as cases with unusual features or confirmed diagnoses are preferentially reported. The limited sample size also precluded meaningful subgroup analyses, including evaluation of which chemotherapy and immunotherapy combinations, targeted therapy responses, and analyses stratified by molecular alterations. Additionally, therapies categorized under treatment groups of chemotherapy and immunotherapy were heterogeneous and included various agents. Further stratified analyses were not feasible due to small sample sizes and inconsistent reporting. The lack of detailed treatment timelines across studies also precluded construction of patient-level visualizations, such as swimmer plots, to assess treatment sequencing and response. Moreover, due to inconsistent reporting of treatment timing relative to diagnosis across included studies, we were unable to perform time-dependent or landmark analyses to account for potential immortal time bias. As such, the observed associations between treatment and survival may be influenced by the fact that patients who survived longer were more likely to receive additional or more aggressive therapies. Finally, baseline clinical variables, including patient demographics, presenting symptoms, or presence of hydrocephalus, were not significantly associated with survival on univariable analysis and were therefore not included in multivariable modeling. However, Residual confounding in the model may persist. Despite this, ours represents the largest integrated analysis of pediatric PDLM cases, offering insight into the overall survival and the impact of different oncologic treatment.

### Future directions

Given the rarity of this disease, meaningful progress in PDLM will require coordinated multicenter efforts. Prospective registries with standardized clinical, imaging, CSF, histologic, and molecular data are needed to better define disease behavior and treatment response. Routine genomic profiling, especially of NRAS and related pathways, may guide eligibility for targeted or combination therapies, including molecularly driven basket trials. Continued study of immunotherapy and intrathecal or systemic chemotherapy within harmonized protocols, alongside exploration of novel targeted and immune-based approaches, represents a key path forward.

## Conclusion

Pediatric PDLM is an exceptionally rare and aggressive leptomeningeal malignancy characterized by diffuse neuroaxis involvement, rapid neurologic decline, and poor survival. In this integrated cohort, immunotherapy and chemotherapy were the only treatment modalities associated with a measurable survival benefit, while radiation therapy did not demonstrate a clear effect. The extreme rarity of PDLM highlights the need for international collaboration, prospective registries, and molecularly informed clinical trials to clarify disease biology and identify effective therapeutic strategies for affected children.

## Data Availability

Data was collected from a systematic search and will be available upon request.
